# Factors Influencing Primary Care Providers’ Unneeded Lumbar Spine MRI Orders for Acute, Uncomplicated Low-Back Pain: a Qualitative Study

**DOI:** 10.1007/s11606-019-05410-y

**Published:** 2019-12-12

**Authors:** Andrea L. Nevedal, Eleanor T. Lewis, Justina Wu, Josephine Jacobs, Jeffrey G. Jarvik, Roger Chou, Paul G. Barnett

**Affiliations:** 1 Center for Innovation to Implementation, VA Palo Alto Healthcare System, 795 Willow Road, Menlo Park, CA 94025 USA; 2 Program Evaluation and Resource Center, VA Office of Mental Health and Suicide Prevention, Menlo Park, CA USA; 3 Health Economics Resource Center, VA Palo Alto Healthcare System, Menlo Park, CA USA; 4 Departments of Radiology, Neurological Surgery, and Health Services, University of Washington, Seattle, WA USA; 5 Department of Clinical Epidemiology and Medical Informatics and Department of Medicine, Oregon Health & Science University, Portland, OR USA

**Keywords:** primary care providers, magnetic resonance imaging, low-back pain, de-implementation, qualitative research

## Abstract

**Background:**

Clinical practice guidelines suggest that magnetic resonance imaging of the lumbar spine (LS-MRI) is unneeded during the first 6 weeks of acute, uncomplicated low-back pain. Unneeded LS-MRIs do not improve patient outcomes, lead to unnecessary surgeries and procedures, and cost the US healthcare system about $300 million dollars per year. However, why primary care providers (PCPs) order unneeded LS-MRI for acute, uncomplicated low-back pain is poorly understood.

**Objective:**

To characterize and explain the factors contributing to PCPs ordering unneeded LS-MRI for acute, uncomplicated low-back pain.

**Design:**

Qualitative study using semi-structured interviews.

**Participants:**

Veterans Affairs PCPs identified from administrative data as having high or low rates of guideline-concordant LS-MRI ordering in 2016.

**Approach:**

Providers were interviewed about their use of LS-MRI for acute, uncomplicated low-back pain and factors contributing to their decision-making. Directed content analysis of transcripts was conducted to identify and compare environmental-, patient-, and provider-level factors contributing to unneeded LS-MRI.

**Key Results:**

Fifty-five PCPs participated (8.6% response rate). Both low (*n* = 33) and high (*n* = 22) guideline-concordant providers reported that LS-MRIs were required for specialty care referrals, but they differed in how other environmental factors (stringency of radiology utilization review, management of patient travel burden, and time constraints) contributed to LS-MRI ordering patterns. Low- and high-guideline-concordant providers reported similar patient factors (beliefs in value of imaging and pressure on providers). However, provider groups differed in how provider-level factors (guideline familiarity and agreement, the extent to which they acquiesced to patients, and belief in the value of LS-MRI) contributed to LS-MRI ordering patterns.

**Conclusions:**

Results describe how diverse environmental, patient, and provider factors contribute to unneeded LS-MRI for acute, uncomplicated low-back pain. Prior research using a single intervention to reduce unneeded LS-MRI has been ineffective. Results suggest that multifaceted de-implementation strategies may be required to reduce unneeded LS-MRI.

**Electronic supplementary material:**

The online version of this article (10.1007/s11606-019-05410-y) contains supplementary material, which is available to authorized users.

## INTRODUCTION

Magnetic resonance imaging of the lumbar spine (LS-MRI) for acute, uncomplicated low-back pain (LBP)^1^ does not improve pain, back function, quality of life, or mental health; however, providers may order imaging anyway.^2–4^ Unneeded LS-MRIs cost the US healthcare system $300 million annually and reveal incidental abnormalities resulting in anxiety,^5^ belief in presence of disease,^2, 6^ and unnecessary procedures.^7–9^ Clinical guidelines suggest acute, uncomplicated LBP should be treated with conservative therapy instead of LS-MRI in the first 6 weeks.^7, 10^ Reducing use of low-value LS-MRI is an important challenge for primary care because LBP is common among adults in the USA.^11^ Prior research found approximately 29% of LS-MRIs in the private sector are unneeded,^12, 13^ with a 22.5% average annual prevalence of this practice nationwide,^14^ and 11% of older patients with acute, uncomplicated LPB receiving unneeded advanced imaging.^15^ Unneeded LS-MRI may be a particular concern for patients seen by the Veterans Health Administration (VA), as more than 10% of patients seen in VA are diagnosed with LBP each year,^16^ and studies using different methods^17, 18^ have found between 30 and 66% of LS-MRI ordered by VA providers were unneeded.

Choosing Wisely targeted LS-MRI in its initiative to reduce low-value services.^19, 20^ Efforts to reduce low-value care have focused on LS-MRI because of strong evidence supporting the clinical practice guidelines and high costs. However, strategies for de-implementing unneeded LS-MRI, including decision support tools,^21^ dissemination,^22^ and audit and feedback,^23^ have not shown lasting effectiveness in experimental and observational studies. Additionally, existing research emphasizes quantitative analyses^24^ of predetermined factors to evaluate unneeded LS-MRI,^24, 25^ but does not adequately identify or explain the various factors contributing to unneeded LS-MRI.

To develop effective de-implementation strategies, PCP perspectives are needed to identify and explain the various factors contributing to unneeded LS-MRI.^26^ This is the first study, to our knowledge, using qualitative methods to elicit VA PCPs’ perspectives to characterize and explain factors influencing unneeded LS-MRI orders for acute, uncomplicated low-back pain and to compare PCPs with low- and high-guideline-concordant ordering patterns.

## METHODS

### Study Design and Setting

As part of a mixed-methods study, semi-structured telephone interviews were conducted with VA PCPs to determine the factors contributing to unneeded LS-MRI for acute, uncomplicated LBP. We used purposeful criterion and random sampling^27^ of high- and low-guideline-concordant providers identified from VA administrative data. Providers were considered low-concordant if ≥ 8% of their index visits resulted in an early scan and high-concordant if < 2% of their index visits resulted in an early scan (Appendix 1). We oversampled low-concordant PCPs. To enhance the transferability of our results, we included diverse PCP types (physicians, nurse practitioners, physician assistants); practice settings (medical centers and community-based outpatient clinics serving various population sizes); and all VA regions.

### Data Collection

We adapted Cabana et al.’s framework^28^ to inform data collection and analysis. We included guideline (familiarity, agreement), patient (treatment preferences), and environmental (time, resources, organizational policies) factors impacting LS-MRI ordering practices. Interview questions explored providers’ practices when treating acute, uncomplicated LBP, including conservative therapy and imaging; knowledge and attitudes about clinical practice guidelines; organizational policies; access to LBP services; and patient preferences (Appendix 2). A PhD-level medical anthropologist designed the interview guide using neutral, open-ended questions, conducted most interviews, and trained three additional interviewers on increasing PCP comfort while discussing sensitive topics, including guideline-discordant behavior. The interview guide was pilot tested with four non-participating PCPs and vetted by an interdisciplinary team.

PCPs were emailed an invitation to participate in a semi-structured telephone interview about LS-MRI for uncomplicated, acute LBP. Follow-up via instant messaging increased response rates.^29^ The Stanford Institutional Review Board approved the study protocol (no. 38033), and participants provided informed verbal consent before interviews. PCPs were blinded to their guideline concordance classification, as this knowledge could bias their responses.

Of the 472 PCPs who were invited, 55 completed interviews, 1 did not complete the interview, and 66 declined. Interviews were audio-recorded and lasted 15–50 min, depending on provider’s availability. Audio recordings were professionally transcribed. Two low-concordant PCPs declined audio recording, so the interviewer took detailed notes instead.

### Data Analysis

Interviewers wrote post-interview analytic memos^30^ using a structured template describing factors contributing to appropriate and unneeded LS-MRI. Provider profiles were summarized in a matrix^31^ to compare factors across high- and low-concordant groups. Two researchers independently coded the same transcripts in ATLAS.ti^32^ software and met weekly to resolve discrepancies.^30^ We performed directed content analysis using an iterative codebook containing deductive factors from Cabana et al.^28^ and other factors identified inductively from interview transcripts.^33^ To better represent our qualitative data, we adapted the Cabana et al.^28^ framework by subsuming guideline factors into a new category named provider factors; this included guideline familiarity and agreement, patient pressure responses, and LS-MRI value. Monthly meetings were held with expert physicians and health economists to discuss complex transcript passages. After completing coding, a primary researcher reviewed the text passages coded in each factor, identified key themes, and summarized results in a comparative matrix. A secondary researcher reviewed the summary for accuracy.

## RESULTS

**Figure 1:**
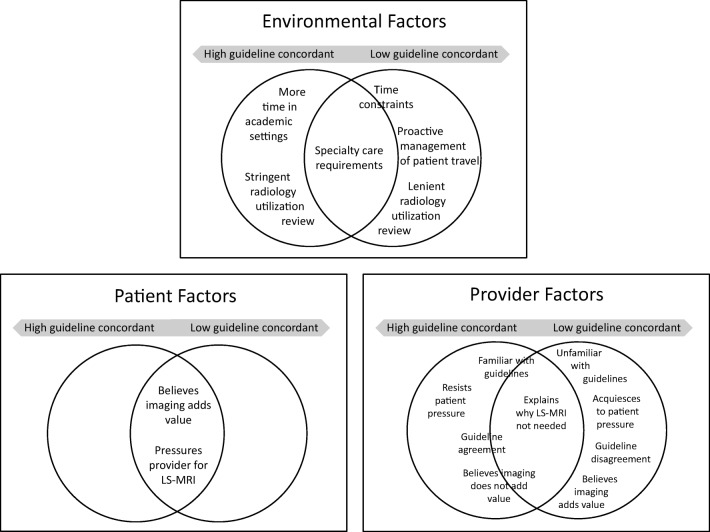
**High and low-guideline-concordant provider groups comparison of environmental, patient, and provider factors influencing unneeded LS-MRI.**

Of the 55 PCPs interviewed, 22 were high and 33 were low-concordant (Table 1). High-concordant PCPs were mostly physicians; however, low-concordant PCPs were divided between physicians and nurse practitioners. Few physician assistants participated. High- and low-concordant providers reported different environmental and provider factors influencing their LS-MRI decision-making and ordering patterns (Fig. 1 and Table 2). However, the high- and low-concordant groups reported similar patient factors.

**Table 1:** Demographics By Provider Group

Primary care providers (*n* = 55)	High-guideline concordance (*n* = 22)	Low-guideline concordance (*n* = 33)
Provider type, no. (%)		
Physician	19 (86)	16 (49)
Nurse practitioner	3 (14)	15 (45)
Physician assistant	0 (0)	2 (6)
Facility type, No. (%)		
VA medical center	8 (36)	11 (33)
Community-based outpatient clinic	14 (64)	22 (64)
Gender, No. (%)		
Female	12 (55)	20 (61)
VA regional service networks*	11 of 18	17 of 18

*All 18 VA geographic regions were represented between high and low-guideline-concordant groups

**Table 2:** High and Low-Guideline-Concordant PCP Responses to Factors Contributing to Unneeded LS-MRI

	High-guideline-concordant PCPs	Low-guideline-concordant PCPs
Environmental factors		
Radiology review	When I order MRIs, the head of radiology calls me to justify it. He wants to automatically cancel it because of the cost. It's frustrating having them canceled by people who have never laid eyes on the patient. To get MRIs approved, I have to show they've done physical therapy, used pain relief, used muscle relaxers. (NP*, CBOC†, #2058)	Before working in VA, I ordered MRI as nurse practitioner, the radiologist had to approve it. Here sometimes they don’t. Maybe if they got more involved in discussing the MRIs [with us] that would be helpful. (NP, CBOC, #3135)
Patient travel burden management	In community clinics, some services are not readily available. You have to work around them, like X-rays and labs. You have to base your diagnosis mainly on clinical [exam] on the first visit. (MD‡, CBOC, #2003)	We had pushback [from radiology] because they felt [LS-MRI and X-ray] shouldn’t be ordered together. We helped them recognize we have patients driving five hours for an X-ray. As Veterans have complained enough, they’ve recognized it won’t hurt to do two services at once. (NP, CBOC, #4030)
Time constraints	We are limited in our time as primary care providers. Finding time to educate patients, for some providers that opportunity is lost because we feel overwhelmed. I try to take time and my patients appreciate that. It makes me feel better at the job I’m doing. Sometimes you have to say, “We’re not able to address other issues.” (MD, CBOC, #6071)	We’re pressed for time, it’s a six-minute walk-in visit, pinched nerve, pain going down leg, I need to know what’s going on and may not have time to elaborate, sometimes it’s easier to order the test. (MD, VAMC§, #3032)
Specialty care requirements	I worked for Department of Defense and we rarely ordered MRIs. It was usually ordered by the anesthesiologist or physiatrist. Here [at VA], it’s completely flipped on its head and it’s done by primary care doctors. (MD, VAMC, #6045)	I inherited patients on narcotics, so when I talk to them about physical therapy, chiropractic treatment, and epidural injections, a lot of them haven't had that. When I'm referring to pain management for epidural injection, you have to order MRI. I ordered a lot because I was getting people off pain meds and treatment they needed. (MD, CBOC, #3089)
Patient factors		
Pressures provider	There is a mindset that every back injury requires an MRI for diagnostic purposes. Patients sometimes pressure providers to request an MRI. (MD*, CBOC, #6192)	[A patient says,] “Look at what they’re getting on the outside. People are getting their backs cured. Why can’t you give me an MRI?” (MD, VAMC, #2039)
Value of imaging	Let's say a patient did not get physical therapy but says, "I know my body. My son had it and physical therapy did not help. I'm not doing physical therapy because I don't see how they can treat somebody when they don't know what the problem is. I want an MRI.” (MD, CBOC, #6055)	A lot of them are not satisfied with a spine X-ray because they want to know is there something that’s hidden that would be revealed with an MRI. (NP, CBOC, #3022)
Provider factors		
Guideline (un)familiarity	We don’t want to stop imaging completely, but we try to image appropriately. I run our lecture series here, and we go over the evidence and then we see what we can do to help patients [with] the tools that we have at VA. (MD, VAMC, #6103)	No, I don’t [rely on guidelines], but when my utilization review nurse says, “You haven’t met this protocol or exhausted other means,” then I’ll stop. I need to be refreshed on the protocol. When you get used to practicing a certain way, you’re like, “do I need it, do I don’t? I’m going to try it and if it goes through, fine. If it doesn’t, then somebody will stop me.” (NP, VAMC, #3047)
Guideline (dis)agreement	I like clinical practice guidelines. It would be good to educate our patients. I think 80 percent of people in their lifetime will have low-back pain and it will resolve within a certain amount of time. (NP, CBOC, #2058)	There’s no criteria for military services and back pain. Our Veterans are unique when it comes to low-back pain. Whether they’re 20 or over 80, even though it may look like no big deal, it could always be something. (NP, CBOC, #4030)
Acquiescing to patients	A guy wanted an MRI now. I said, “What have you done to get better?” He had done nothing. I said, “If you’re an NFL quarterback who sustained a big hit, they might do an acute MRI right now. For the rest of us, that’s not the guideline.” I share evidence-based stuff with them. Generally, guys are agreeable. (MD, CBOC, #6158)	From the patient’s perspective, they are paranoid there’s something missing until we do the MRI. We tell them there is nothing that can be surgically corrected. They want that final step. I don’t know if you listen to the patients or you listen to the guidelines, but if you’re trying to help the patient, that’s who we have to follow, not the guidelines. (MD, CBOC, #3113)
Value of imaging	If there are no red-flags, no reason to think that I need to send this patient on for some intervention, I’m not ordering an MRI. I’m only ordering if there are some red-flag symptoms and I’m going to send them to a neurosurgeon. I look at MRI as a preliminary workup for some invasive procedure. An orthopedic surgeon once told me, “Why draw a map if you’re not ready to take the trip?” (MD, VAMC, #2077)	If there’s been trauma involved, even though there’s no red-flags, I think it’s worthwhile. Sometimes their complaints are vague, or you’re not sure if it’s something else, so there’s value in that. (NP, CBOC, #3015)

*NP, nurse practitioner

^†^*CBOC*, VA community-based outpatient clinic

^‡^*MD*, physician

^§^*VAMC*, VA Medical Center

### Environmental Factors Contributing to Unneeded LS-MRI

#### Lenient Radiology Utilization Review

Low-concordant providers reported more autonomy when ordering LS-MRI, and few discontinued LS-MRI orders (“If we feel that’s what needs to be done, they don’t give us any issues.”), which contributed to unneeded LS-MRI. High-concordant providers reported a more stringent radiology utilization review process. For LS-MRI approval, providers documented the patient’s 6-week conservative therapy use (e.g., medication management, physical therapy), completed an order appropriateness template, and/or contacted radiology to determine the order’s appropriateness (“The MRI cannot be done unless you call the radiologist”). High-concordant providers described higher rates of LS-MRI discontinuation, more decision support tools, narrower criteria for approving LS-MRI, and less autonomy, which reduced unneeded LS-MRI.

#### Proactive Management of Patient Travel Burden

Low-concordant providers emphasized how patient access challenges influenced imaging decisions. When patients lived far away from imaging and specialty care, low-concordant providers adopted a proactive approach. PCPs “put the [LS-MRI] order in at the same time as the X-rays” and specialty care consult. Low-concordant PCPs explained how this proactive approach was initially rejected by their radiology utilization review, but later accepted to reduce patient travel burden. PCPs mentioned that LBP might resolve before the scan was available. High-concordant providers did not discuss altering imaging decisions for patients travelling long distances for care.

#### Time Constraints During Patient Visits

Low-concordant providers described how time constraints during clinical appointments, especially for “walk-in” patients, were a frequent contributor to unneeded LS-MRI. When experiencing time constraints, low-concordant providers used LS-MRI instead of a thorough history and physical because “it is easier to order [LS-MRI] than to sit and talk with everybody or do the follow-up that’s needed.” High-concordant providers did not discuss time constraints impacting LS-MRI but thought “time is the biggest barrier to educating the patient.” High-concordant providers working in VAs affiliated with academic teaching facilities reported more time with patients, which fostered in-depth examinations and discussions about the appropriateness of LS-MRI.

#### Specialty Care Requirements

High- and low-concordant PCPs stated LS-MRI was required for some specialty care referrals. Providers sometimes thought LS-MRI were unnecessary but ordered them, so specialty care clinics would accept referrals (“I don’t know why pain management requires it”). Although providers thought LS-MRI were required for referrals to specialty care, one high-concordant provider noted that specialty care might expect LS-MRI but not require it; therefore, inaccurate expectations could contribute to imaging overuse.

### Patient Factors Contributing to Unneeded LS-MRI

#### Beliefs in the Value of Imaging and Pressure on Providers

Both PCP groups thought patients believed in the added value of imaging, which resulted in patients pressuring their providers for unneeded LS-MRI. PCPs stated that patients believe “imaging is the answer” to back pain, noting these patient beliefs reflected knowledge gaps about appropriate acute LBP treatment, how long it takes acute LBP to resolve, limited utility of LS-MRI for treating LBP, and conservative therapy options as first-line treatment. Providers emphasized that patient pressure for unnecessary LS-MRI is an on-going challenge.

### Provider Factors Contributing to Unneeded LS-MRI

#### Limited Guideline Familiarity

Low-concordant providers were less familiar with guidelines than high-concordant providers. Some low-concordant providers sought information about imaging guidelines and were unaware of existing guidelines created by VA and the Department of Defense (e.g., “If the VA presents specific guidelines of ‘this is what you need to do for MRIs,’ then that would be better for us, because then we wouldn’t have to decide, is this an MRI case or is this not an MRI case?”). Among low-concordant providers who were familiar with guidelines, their knowledge was sometimes negated by competing factors, described next. High-concordant providers emphasized “practicing evidence-based medicine” and reported more knowledge of imaging guidelines and back examinations to identify red-flag conditions. Some high-concordant providers described themselves as educators and experts on LS-MRI guidelines.

#### Guideline Disagreements

Disagreement with LS-MRI guidelines was present although not pervasive among the low-concordant group, but not expressed by high-concordant providers. Low-concordant providers who disagreed with guidelines believed Veterans should be imaged more often because they were high-risk, and guidelines were less relevant to their Veteran population, who presented with “yellow flag issues that are not necessarily in evidence clinically.”

#### Acquiescing to Patient Pressure

Patients pressured both PCP groups for LS-MRI, but PCP groups had different responses. Low-concordant providers acceded to patient requests for LS-MRI, despite being familiar with guidelines, explaining why a LS-MRI was not needed, and knowing LS-MRI was unnecessary (“Sometimes patients who threaten to go to the patient advocate can motivate you to do things you wouldn’t otherwise do”). Low-concordant providers worried about patient retaliation or ordered LS-MRI to maintain relationships with patients. High-concordant providers resisted patient pressure, discussed evidence-based alternatives, and explained why LS-MRI was unneeded (“I stick to evidence-based medicine. I don’t try to make patients happy by complying with their wants”).

#### Beliefs in the Value of Imaging

Low-concordant providers thought LS-MRI had some value for treating acute, uncomplicated LBP (“Imaging is sometimes therapeutic [for patients], the pain will go away after the test is done”). Rationales included to avoid missing something, to establish a new patient’s baseline, to determine patient eligibility for pain injections (e.g., to improve sciatica symptoms or reduce opioid use), to determine the cause of sudden high pain scores, and to increase access to specialty care. High-concordant providers did not describe added value and did not provide rationales for ordering LS-MRI for acute, uncomplicated LBP (“[If there’s] no difference in management, [then] why do an MRI?”). High-concordant PCPs reported only ordering LS-MRI for acute, uncomplicated LBP when it was required for specialty care referrals.

### Provider Recommendations for Reducing Unneeded LS-MRI

#### Improve PCP Guideline Knowledge and Utilization Review

Providers suggested several strategies to improve their clinical knowledge and skills and enhance their clinical tools: more protected time for educational opportunities; skills training on managing patient pressure; brief in-services from specialists on imaging guidelines and back exam techniques; decision support tools; and closer collaboration with radiologists.

#### Enhance Access to Alternatives

Providers wanted improved access to LS-MRI alternatives for their patients. Providers recommended same-day primary care access to a brief session with a physical therapist as an alternative to LS-MRI, especially for patients with trouble accessing traditional physical therapy. Providers also suggested improving patients’ access to complementary and integrative health treatments (e.g., chiropractic, yoga, massage, acupuncture); gyms; swimming pools; pain injections; medications (e.g., lidocaine patches); and medical equipment (e.g., massage tools).

#### Increase Time with Patients

Providers wanted more time to take a detailed history, perform a thorough back exam, and discuss patient preferences and guidelines. PCPs suggested protecting time or designating a provider for “walk-in” patients.

#### Boost Patient Guideline Awareness

PCPs wanted patients to receive education about clinical guidelines outside of the exam room. Providers’ suggestions included educational materials for patients to take home, videos on back pain and imaging in waiting areas, handouts on when imaging is appropriate or unneeded, and information on pain management exercises and mind-body techniques in clinical areas.

## DISCUSSION

LS-MRI is not recommended for acute, uncomplicated low-back pain prior to 6 weeks of conservative therapy. However, some VA PCPs order LS-MRI anyway, and their reasons for these ordering patterns have not been well understood. We identified and characterized environmental, patient, and provider factors contributing to unneeded LS-MRI, many of which differed between low and high-concordant PCP groups. Using qualitative methods was a novel approach to identifying factors contributing to unneeded LS-MRI. Our results enhance existing literature^34–37^ by explaining why low- and high-concordant PCPs differ in their use of unneeded LS-MRI for acute, uncomplicated LBP. Our results highlight diverse factors contributing to unneeded LS-MRI, suggesting that de-implementation efforts should incorporate multifaceted strategies rather than a single strategy.

Prior research, including guideline dissemination,^22^ decision support tools, provider counseling,^38^ and audit and feedback,^39^ provides limited evidence on the efficacy of single interventions in reducing unneeded LS-MRI. ^21, 23, 40^ Single interventions do not account for how multiple barriers interact and affect overuse of LS-MRI. Although PCPs supported interventions targeting provider guideline knowledge, we found that such knowledge can be negated by other factors (e.g., patient pressure, guideline disagreement). For example, some low-concordant PCPs imaged Veterans more often because they thought clinical guidelines did not account for Veterans’ unique needs; this supports research on how provider guideline concordance is affected by perceptions of applicability to patients.^35^ Although the VA has Veteran-specific imaging guidelines, some providers are unaware of them.^41^ Providers who are familiar with guidelines, but still have low compliance, may require interventions other than knowledge-based ones to change behavior, such as understanding healthcare costs of unneeded scans or improving access to treatments such as physical therapy.

New recommendations for audit and suggestive feedback^42^ could inform providers of conservative therapy options. However, providers described a lack of these services and wanted more same-day alternatives (e.g., physical therapy, massage) to help reduce overuse of LS-MRI and improve LBP care. Research has shown that Veterans use other services when available,^43^ and chiropractic care is a viable option at no additional expense.^44^ Research is needed to determine if increasing access to same-day alternatives, even if they do not have strong clinical evidence, helps reduce unnecessary LS-MRI.

While our findings align with research showing that radiology utilization review may reduce inappropriate MRI ordering in the short term,^45, 46^ radiologists may not have the time or desire to function as gatekeepers.^47^ Research is needed on the feasibility of implementing and sustaining radiologist utilization review on a large scale.^45^

PCPs thought time constraints contributed to unnecessary LS-MRI, which is in line with existing literature on low-value care.^48^ However, extra time requires administrative support and resources and may not always be feasible. Research is needed to understand if there is any impact on unneeded LS-MRI ordering when patients have longer appointments, including effects of performing a thorough history and physical exam.

Both high- and low-concordant PCPs thought requiring LS-MRI for certain specialty care referrals was sometimes unnecessary. Additional research would help determine if specialty care requirements contribute to LS-MRI overuse and if low-concordant PCPs overuse specialty care.

PCPs emphasized how patients believe imaging adds value and will insist on LS-MRI because they are unaware of or cannot accept guidelines. We expand others’ research about the role of patient guideline acceptance in LS-MRI ordering^24, 34, 36, 37^ by showing how high- and low-concordant providers respond differently to patient pressure. Providers thought patients would benefit from educational materials on imaging guidelines and LBP, but research is needed to understand optimal patient education content, delivery methods, and effects on utilization.^49^

Comparing high- and low-concordant PCP groups provides insight into causes for unneeded LS-MRI orders. High-concordant providers demonstrate clinical guideline best practices. Low-concordant PCPs describe factors contributing to unneeded LS-MRI and highlight topics underrepresented in research on reducing low-value care. For example, low-concordant providers emphasized sensitivity to patient needs; these PCPs ordered LS-MRI to maintain patient relationships and reduce travel burden. Research^50^ and clinical guidelines do not discuss ordering LS-MRI to address patient preferences and needs. Yet, this raises questions about the role of patient perceptions in LS-MRI overuse and how to incorporate patient preferences and needs when de-implementing low-value care to improve health equity, which could be explored in future research.^51^

This qualitative study has limitations. First, our study focuses on PCP perspectives which may not reflect radiologist and patient perspectives that could also provide information on unneeded LS-MRI. In the VA, PCPs are the driver of unneeded LS-MRI,^17^ which is why they were the focus of this study. Second, since our study is VA-based and Veterans may have unique needs, some results may differ from other healthcare systems. For example, VA PCPs did not mention financial incentives as factors influencing their LS-MRI ordering decisions.^52, 53^ Incentives for ordering LS-MRIs may differ from non-VA settings because VA PCPs do not receive monetary gain when ordering more LS-MRIs. In addition, VA PCPs’ actions are insured by the federal government against malpractice claims.^54^ Although some low-concordant providers mentioned ordering LS-MRI to avoid missing something, they generally did not make statements suggesting they practice defensive medicine. Third, PCPs with guideline-discordant practices might be less willing to participate or disclose their behavior. To combat this potential issue, PCPs were not aware of their guideline-concordant status, their responses were confidential, administrative data were used to identify providers with low-concordant behavior, and we oversampled this group.

Depending on local context, some factors contributing to unneeded LS-MRI may be more modifiable than others. A multifaceted approach, including offering same-day alternatives, may be required for effective, long-lasting de-implementation of unnecessary LS-MRI. Our results enhance knowledge about factors leading to unneeded LS-MRI, which may inform the VA Choosing Wisely Committee and other healthcare systems in developing multifaceted interventions to reduce unneeded LS-MRI for acute, uncomplicated low-back pain and low-value care overall.

## Appendix

ESM 1 (DOCX 32.0 KB).
