# Supplementary material for: Factors Influencing Primary Care Providers’ Unneeded Lumbar Spine MRI Orders for Acute, Uncomplicated Low-Back Pain: a Qualitative Study
Source: J Gen Intern Med. 2019 Dec 12;35(4):1044–51. doi: 10.1007/s11606-019-05410-y (PMC7174262; doi:10.1007/s11606-019-05410-y)
Supplement: ESM 1 — (DOCX 32.0 KB). [file 11606_2019_5410_MOESM1_ESM.docx]

**Appendices**

**Appendix 1. Description of High and Low-Guideline-Concordant Primary Care Provider Cohorts Identified From Administrative Data**

To identify high and low-guideline-concordant primary care providers, we first created a database of uncomplicated low-back pain index visits using the VA’s Outpatient National Patient Care Database SE file. Uncomplicated low-back pain cases excluded complex cases where imaging may be appropriate, so visits where the patient had a clinical diagnosis for spinal stenosis, radiculopathy, sciatica, or lumbago were excluded. We also excluded individuals who had history of the following red-flag conditions: spinal surgery (look-back of 12 months), infectious conditions (look-back of 12 months), congenital spinal conditions (look-back of 5 years), autoimmune disorders (look-back of 5 years), spinal cord infarction (look-back of 12 months), cancer (look-back of 5 years), recent trauma (look-back of 90 days), IV drug abuse (look-back of 12 months), and neurological impairment (look-back of 12 months)^54^ . Visits were also excluded if the patient had a recent history of low-back pain (i.e. prior outpatient visits with a diagnosis for lumbar or thoracic back pain in the 12 months preceding the index visit).

The Managerial Cost Accounting Radiology file and the fee basis outpatient visits file (i.e. the contracted care file) were then used to identify encounters where the patient received a lumbar spine MRI within 42 days of the index visit. The 42 day period was selected based on clinical guidelines, which recommend that clinicians avoid imaging within the first 6 weeks of acute uncomplicated low-back pain episodes, unless red-flag conditions are present^20^ . The Primary Care Management Module (PCMM) database was used to identify the assigned primary care providers at the time of the index visit; if the patient had not assigned primary care provider, the visit was deemed to be the responsibility of the primary care provider seen in the index visit. We identified providers who had at least 10 visits between 01/01/2015 and 06/30/2016, resulting in 6,486 providers. We then used the Corporate Data Warehouse (CDW) Staff table to exclude providers who were not nurse practitioners, physicians, resident physicians, or physicians’ assistants. The VA Planning Systems Support Group (PSSG) file was used to identify provider-level practice location to identify whether the provider practiced at a hospital or a clinic. Finally, we limited the cohort to providers who had at least 20 index visits and could be characterized as a low-concordant or a high-concordant provider. Providers were considered low-concordant if ≥8% of their index visits resulted in an early scan and high-concordant if <2% of their index visits resulted in an early scan. These thresholds were selected from the observed distribution to generate a sufficient sample to allow recruitment.

**Appendix 2. Abridged Interview Guide**

1. To begin, please walk me through your approach to the initial diagnosis and treatment for acute low-back pain without any red-flag conditions.
2. Tell me about your experience with providing conservative therapy for patients with acute low-back pain without red-flag conditions.
3. What factors do you consider when you decide to order a LS-MRI for acute low-back pain without red-flag conditions?
4. Do you order LS-MRI for acute low-back pain without red-flag conditions? Why/why not?
5. What challenges/barriers do you experience when treating acute low-back pain?
6. Why might a provider offer LS-MRI for acute low-back pain without red-flag conditions?
7. Walk me through the process of ordering a LS-MRI for acute low-back pain without red-flag conditions at your facility.
8. For acute low-back pain without red-flag conditions, are you ever required to order LS-MRI for referrals to specialty care?
9. Do you rely on guidelines/resources in deciding whether to order an MRI? Why/why not?
10. Do patients ever request LS-MRI for acute low-back pain without red-flag conditions? How do you handle this?
11. What support and resources do you need to improve care for acute low-back pain?
12. Anything else that you would like to add to our conversation today?
